# A Scoping Review of Intimate Partner Violence Research in Canada

**DOI:** 10.1177/15248380241275979

**Published:** 2024-09-13

**Authors:** C. Nadine Wathen, Jennifer C.D. MacGregor, Caitlin Burd, Najibullah Naeemzadah, Yetunde A. Ogunpitan, Jaimeson Canie

**Affiliations:** 1 Professor & Canada Research Chair in Mobilizing Knowledge on Gender-Based Violence, Arthur Labatt Family School of Nursing, Canada; 2 Western University, Canada

**Keywords:** cultural contexts, domestic violence, intervention/treatment

## Abstract

Intimate partner violence (IPV) is at epidemic levels across low-, middle-, and high-income countries, including Canada, where recent lifetime prevalence indicated that over 40% of women had IPV experiences. In response to this, Canada’s federal government has made investments toward IPV prevention and response. We conducted a scoping review of English and French literature identified through searches of multiple databases and specific journals to assess the current state of IPV research in Canada. A total of 267 articles met inclusion criteria of being peer-reviewed research primarily about IPV in either French or English published from 2020 to 2022 with at least one Canadian-affiliated author. Almost a third of studies described services for survivors but did not evaluate service effectiveness. We noted a significant gap in research on the IPV experiences of gender and/or sexual minorities. Canada’s federal social science research funding agency was the most common funder, with the two federal government departments with specific IPV funding initiatives in place cited as funding less than 6% of included studies. In general, there remains an overfocus on IPV epidemiology and on descriptions of service use, and not enough research examining the effectiveness and implementation of interventions, especially grounded in theoretical, gendered, and trauma- and violence-informed frameworks. Funders and researchers are encouraged to consider moving resources from ongoing description of well-established factors to assessment and implementation of evidence-informed interventions, and, crucially, primary prevention of IPV and all forms of gender-based violence.

Intimate partner violence (IPV) is “behaviour within an intimate relationship that causes or has the potential to cause physical, sexual, or psychological harm, including acts of physical aggression, sexual coercion, psychological abuse, and controlling behaviours” (World Health Organization [WHO], 2010) and is the most prevalent form of gender-based violence (GBV). The WHO (2021) declared IPV a public health crisis with global data from 2018 indicating that 27% of women experience IPV in their lifetime and 13% in the past year (Sardinha et al., 2022). In Canada, the rates are even higher, with a 2018 nationally representative cross-sectional survey of over 45,600 people finding that 44% of women who have been in an intimate relationship report experiencing lifetime IPV (Government of Canada, 2022). The health and social consequences of IPV on survivors, especially women, and society are well-documented (Bacchus et al., 2018), including poorer physical and mental health, increased health risk behaviors (Bonomi et al., 2009; Devries et al., 2013; Devries et al., 2014), harm to children experiencing IPV (Bair-Merritt et al., 2006), and very large economic costs (Ashe et al., 2016; Duvvury et al., 2013). IPV was made significantly worse by the COVID-19 pandemic (Leight, 2022; UN Women, 2021) and pandemic policy responses (Wathen et al., 2022), leading to its framing as a “shadow pandemic” (Mantler, Wathen et al., 2022), and more recently, the recognition, including through legislation, by multiple levels of governments in Canada (federal, provincial, and municipal) of IPV as itself an “epidemic.”

Canada has made significant investments to develop and evaluate programs and services for IPV prevention and response. Some examples include the Public Health Agency of Canada’s $100 million investment in 2014/2015 to support the health and well-being of survivors of family violence (Government of Canada, 2015), and the 2017 Federal GBV Strategy (also called *It’s Time: Canada’s Strategy to Prevent and Address Gender-based Violence*) developed by Women and Gender Equality (WAGE) Canada. The latter provides $800 million in funding, with $44 million annually, for preventing GBV, providing support to survivors and their families, and improving the justice system’s responsiveness (Government of Canada, 2023b). Most recently, the Canadian federal government, across multiple departments, has committed to investing in the National Action Plan to End GBV, which builds on current federal, provincial, and territorial government efforts and intends to address the root causes of violence across and within regions in Canada (Women and Gender Equality Canada, 2022a). These activities include core funding for research to develop evidence in support of prevention, intervention, and policy development. For example, advocates and agencies in the anti-violence sector engaged in extensive research and consultation to develop a roadmap to guide the creation and implementation of the National Action Plan (Dale et al., 2021). A separate National Action Plan specific to Missing and Murdered Indigenous Women, Girls, and 2SLGBTQQIA+ People was also federally funded (Government of Canada, 2023a)with a report published in 2021 (Core Working Group, 2021). However, no existing review has examined Canadian IPV research.

In general, since an early systematic review (Wathen & MacMillan, 2003) identified a paucity of intervention research to inform policy and practice, the amount of worldwide IPV research has grown significantly. A July 2023 Scopus (federated database) search of the terms (“intimate partner violence” OR “domestic violence”) has yielded about a thousand (987) review articles since 2003 (and over 12,700 articles when removing the review filter). In addition, international agencies, such as the World Health Organization and UN Women, have established standards, funding opportunities and repositories to enable better data collection and reporting and synthesize findings in support of systemic and multi-level change.^1^

Given the recent rise in investments in IPV research and response in Canada, it is important to examine research outputs on this topic to articulate what is being done and what gaps may persist. As such, we conducted a scoping review of Canadian IPV research. Systematic review and synthesis methods of a given research topic in a specific geographic region have been used to understand the extent and nature of research activity and also how this activity relates to contextual factors, such as gauging how a topic is funded, prioritized and, in some cases, relevant to specific practice or policy needs (Bradbury-Jones et al., 2019; Nagi et al., 2020; Osborne et al., 2020). For example, Bradbury-Jones et al. (2019) conducted a mapping review and synthesis to produce a profile of the nature and scope of European GBV research. These authors focused on studies published in specific journals during a one-year period and required at least one author to be Europe-affiliated. In an unrelated field, Nagi et al. (2020) conducted an environmental scan to examine Canadian expertise in global health research focusing on (a) funding, (b) ongoing research activities (e.g., training programs and research centers), and (c) published research. Finally, Osborne et al. (2020) used a series of rapid reviews to identify potential policy interventions specific to social issues affecting rural Australians.

Therefore, to better understand the specific landscape of IPV research in Canada, we conducted a scoping review to answer the following key questions: (a) What areas of IPV research are being studied versus neglected? (b) What research methods are being used? (c) Where and by whom is the research being done? (d) By whom is it funded? We also noted new innovative topics or approaches addressing specific research gaps.

## Method

### Overview

A systematic scoping review is well-suited for “mapping” existing research and highlighting research gaps (Arksey & O’Malley, 2005; Daudt et al., 2013). Because we were interested in describing the state of IPV research in Canada in particular, we were guided by the work of Bradbury-Jones et al. (2019), who conducted a review of GBV research in Europe.

### Search Strategies

Similar to Bradbury-Jones et al.’s (2019) method, to capture a snapshot of recent research in Canada, our searches were limited to the years 2020 to 2022^2^; this time period would likely include research activities spurred by the government investments discussed above, providing adequate time for studies to be undertaken and published. Our primary search strategy involved hand searching 11 violence-focused journals identified by Bradbury-Jones et al. as the highest ranked in the field. Specifically, four team members (two per journal) independently viewed the online tables of contents of the journals and placed article reference information into a spreadsheet if the article, based on its title, abstract, and author affiliations (if available) appeared to meet inclusion criteria (see below). The journals were: *Child Abuse & Neglect; Sexual Abuse; Journal of Elder Abuse and Neglect; Child Abuse Review; Trauma, Violence & Abuse; Journal of Aggression, Maltreatment & Trauma; Journal of Interpersonal Violence; Violence Against Women; Violence and Victims; Journal of Family Violence;* and *Psychology of Violence*. We excluded two journals from Bradbury-Jones et al. that were unlikely to include IPV-focused articles: *Child Sexual Abuse* and *Journal of School Violence*. To supplement our hand search, the second author conducted a focused search of Scopus using the terms “domestic violence” or “intimate partner violence” in the title or abstract, and “Canada” as the country of author affiliation. To capture French-language articles specifically, a bilingual team member conducted focused searches using the equivalent French terms in two databases: Cairn and Érudit. All searches were conducted in November 2022. Finally, forward and backward citation chaining to identify cited and citing articles was conducted by two team members in April and May 2023.

### Screening and Eligibility

All hand searches (which functioned as title/abstract-level screening) were done independently by two team members. The full text of articles identified as potentially relevant was screened independently by two team members, a third resolved any disagreements, and the first author was consulted for difficult decisions. Search results from Scopus were screened at the title and abstract level first using the online software Rayyan (Ouzzani et al., 2016). When in doubt, articles were moved forward to full text review and the same decision process as above for articles located through the hand search was used. Only unique articles from Scopus (i.e., not ones that had already been assessed during the hand search phase) were screened. Articles published in French were assessed by a fully bilingual team member and discussed with another team member to confirm inclusion decisions.

Peer-reviewed research articles that were primarily about any form(s) of IPV (according to the WHO, 2010 definition provided above) and published in either official language of Canada (French or English) from 2020 through 2022 with at least one Canadian-affiliated author were eligible for inclusion. Articles focused on other topics, such as dating violence, elder abuse (outside of intimate relationships), sibling violence, and child maltreatment (including children’s exposure to IPV), were excluded. Articles on broader topics, such as violence against women, GBV, or family violence, were included if there was specific attention to IPV. Primary research articles (as well as the secondary analysis of an existing dataset, e.g., national-level data, data in the public domain) were further required to include at least some Canadian data, whether from participants or other sources (e.g., news media or policy documents) or, if not specified, to be reasonably expected to include some Canadian data (e.g., a study of Reddit or Twitter posts with at least one Canadian-affiliated author). Evidence synthesis articles (i.e., systematic reviews, multi-study meta-analyses or other types of reviews) were eligible for inclusion; however, due to the dearth of Canada-specific reviews, they were not required to include Canadian sources. Commentaries, research protocols, and overviews (i.e., summaries of evidence with no methodological details reported) were excluded. Given the goal of this review to describe the nature of Canadian IPV research, and standard practice in scoping reviews (Peters et al., 2015; Pham et al., 2014), we did not undertake quality assessment of the included articles.

### Data Extraction, Coding, and Analysis

All data (e.g., sample characteristics, study design, etc.) from included articles were extracted by one team member, entered into a spreadsheet, and verified by a second team member. The four team members involved in screening made notes regarding the main article topics they observed; these preliminary themes were consolidated by the second author and reviewed by the other team members to develop a coding scheme. The article topic codes were then applied to each article during the data extraction process (articles could receive a maximum of three topic codes) and verified, with disagreements resolved by a third team member. Quantitative analyses of all article characteristics using descriptive statistics were performed using SPSS Version 28 (IBM, Aramok, NY).

## Results

### Overview

**Figure 1.**
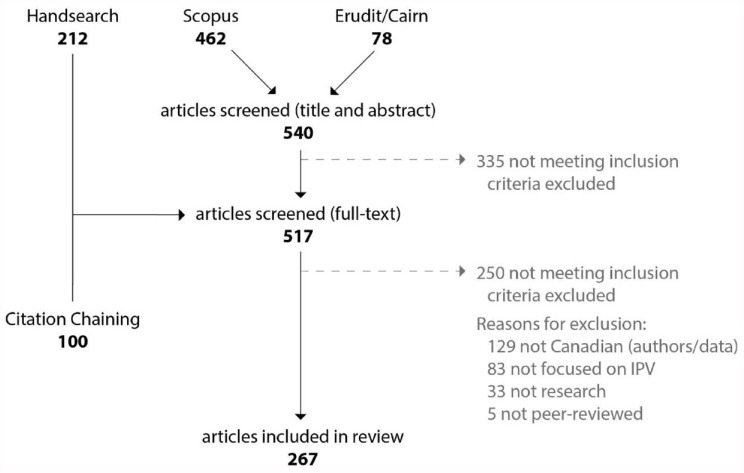
Search flow diagram. *Note.* Numbers in bold represent # of unique articles identified (i.e., after duplicates removed).

In total, we screened 517 full-text articles, and 267 articles meeting our inclusion criteria were included in this review (Figure 1). Some included articles reported findings from the same study, but our unit of analysis was the articles themselves. Most articles were published in English (*n* = 255; 95.5%; French, *n* = 12; 4.5%). The number of articles increased with each publication year (2020: *n* = 51, 19.1%; 2021: *n* = 98, 36.7%; 2022: *n* = 118, 44.2%). Most articles were published in social science journals (*n* = 202, 75.7%), with the remaining in health-related journals (*n* = 63, 23.6%) and other types (*n* = 2, 0.7%). Roughly half were published in a violence-specific journal (*n* = 139, 52.1%).

### Areas of Research

Table 1 presents findings regarding the article topics as well as examples of articles receiving each topic code. Most articles received two topic codes (*n* = 141, 52.8%; *M* = 2.0, *SD* = .69). The most common article topics across all included articles were IPV (and related) services (e.g., barriers to IPV services and ways to improve, how providers such as physicians deliver care to IPV survivors, and provider and survivor experiences of care) and IPV and psychological factors (e.g., mental health correlates of surviving or perpetrating IPV, psychological outcomes of IPV treatment programs, and personality correlates of IPV perpetration). Nearly a quarter of all included articles received the “other” code, which represented topics that were not frequent enough to warrant the creation of a new code. Some of these topics included IPV and work or the workplace, IPV discourses in (social) media, knowledge translation, informal supports, empowerment, and concerns related to survivors’ pets and/or livestock. Among review articles (*n* = 41; 15.4%), the most common topics were IPV interventions (*n* = 11, 26.8%), “other” (see explanation above; *n* = 12, 29.3%), IPV (and related) services (*n* = 11, 26.8%), and physical health (*n* = 8, 19.5%). For all articles, the most common “specific group” topic (i.e., a group that may not typically receive research attention) was men as perpetrators, followed by men as survivors. The most common specific “types” of IPV focused on across all articles were femicide/homicide (including attempted femicide/homicide) and sexual violence.

**Table 1.** Frequency of Article Topic Codes with Example Articles.

Code	Articles Receiving Code *n* (%)	Example Article Receiving Code^a^
Specific groups		
Men as perpetrators	25 (9.4)	Léveillée et al. (2022)
Men as survivors	18 (6.7)	Brooks et al. (2020)
Immigrant/newcomers	13 (4.9)	Park et al. (2021)
Rural/remote communities	12 (4.5)	Mantler et al. (2020)
Indigenous people	11 (4.1)	Varcoe et al. (2021)
2SLGBTQ+	5 (1.9)	Roy et al. (2022)
Older adults	3 (1.1)	Weeks et al. (2021)
Veterans	2 (.8)	Cowlishaw et al. (2022)
Specific types of IPV^b^		
Femicide/homicide (including attempted)	14 (5.2)	Saxton et al. (2022)
Sexual	14 (5.2)	Jung et al. (2021)
Pet abuse	8 (3.0)	Giesbrecht (2022)
Physical	7 (2.6)	MacDonald et al. (2021)
Psychological/emotional	6 (2.3)	Dugal et al. (2020)
Financial	2 (.8)	McKay White and Fjellner (2022)
Other (e.g., stalking, familicide)	6 (2.3)	Myers et al. (2021)
Other		
IPV (and related) services	82 (30.7)	Baird et al. (2021)
Psychological factors (e.g., personality traits)	55 (20.6)	Plouffe et al. (2022)
IPV interventions^c^	37 (13.9)	Montesanti et al. (2022)
Legal/justice system	37 (13.9)	Sheehy and Boyd (2020)
Physical health (e.g., traumatic brain injury)	32 (12.0)	Haag et al. (2022)
COVID-19 and IPV	27 (10.1)	Toccalino et al. (2022)
Motherhood/fatherhood/parenting	23 (8.6)	Song-Choi and Woodin (2021)
Child maltreatment	16 (6.0)	Brassard et al. (2020)
IPV measurement and scale validation	8 (3.0)	Côté et al. (2021)
IPV prevalence and severity	7 (2.6)	Mojahed et al. (2021)
IPV risk assessment	5 (1.9)	Hilton and Radatz (2021)
Other	59 (22.1)	Cameron et al. (2020); MacGregor et al. (2022); Sivagurunathan et al. (2021)

*Note.* IPV = Intimate partner violence.

a Due to page constraints, only example articles are provided; a table specifying the codes applied to all articles is available upon request.

b While many articles may have reported on specific types of IPV, only those with a focus on better understanding a particular type received these codes.

c Broader than the finding reported in main text regarding evaluating interventions.

### Research Methods

Table 2 presents the study characteristics described in the included articles. The vast majority of articles reported research that was not clearly influenced by, or based in, a particular theory (*n* = 184, 68.9%). Of the articles clearly stating their theoretical approach (*n* = 83, 31.1%), the most common were various forms of feminist theory (*n* = 31, 37.4%; e.g., intersectional feminism, feminist ethical theory, etc.), grounded theory (*n* = 13, 15.7%; including constructivist theory), and intersectionality (excluding intersectional feminism; *n* = 12, 14.5%). While most of the included articles reported the results of quantitative research, qualitative research was a close second. Relatively few articles reported the results of intervention research (*n* = 33, 12.4%); of these, 10 were mixed-method studies (30.3%), 10 were quantitative, 8 were reviews (24.2%), and 5 were qualitative studies (15.2%). Among the research articles reporting findings from original analyses of primary or secondary data (non-review papers, *n* = 226), 58.4% (*n* = 132), measured (i.e., collected information about it in some way, including coding from existing data) IPV victimization or perpetration (e.g., frequency, severity) or a closely related variable (e.g., risk of IPV). Of these, 51.5% (*n* = 68) used or adapted an established measurement tool; the most common were the Conflict Tactics Scale (CTS) (*n* = 27, 39.7%) and the Composite Abuse Scale (*n* = 11, 16.2%), including different versions and short forms of each (Ford-Gilboe et al., 2016; Hegarty et al., 2005; Straus, 1979; Straus & Douglas, 2004). Most other tools used across the 68 articles that specified instrument use were used in three or fewer articles each. Articles that did not measure IPV^3^ often involved samples of survivors or perpetrators who were self- or provider-identified, or service providers themselves who were asked about their work experiences or their perspectives of survivor experiences, for example. Among non-review articles, most samples included at least some IPV survivors and/or perpetrators (*n* = 150, 66.4%; see Table 2 for more sample characteristics). Only 23.6% of articles overall reported including any gender and/or sexual minority people in their sample. Not surprisingly, given the prevalence of IPV exposure in women, among articles focused on survivors (*n* = 91), most only included women (including trans women; *n* = 66, 72.5%).

**Table 2.** Characteristics of Research in Included Articles (*N* = 267).

Characteristic	*n* (%)
Study type/design^a^	
Quantitative (*n* = 104; 39.0%)	
Cross-sectional	42 (40.4)
Longitudinal	37 (35.6)
Other/multiple designs (e.g., scale validation, pre-post)	23 (22.1)
Unclear/not specified	2 (1.9)
Qualitative (*n* = 90; 33.7%)	
Cross-sectional	17 (18.9)
Grounded theory	6 (6.7)
Other/multiple designs (e.g., photovoice, ethnography)	36 (40)
Unclear/not specified	31 (34.4)
Mixed (*n* = 32; 12.0%)	
Cross-sectional	15 (46.9)
Other/multiple design (e.g., pre-post, longitudinal)	12 (37.5)
Unclear/not specified	5 (15.6)
Review (*n* = 41; 15.4%)	
Systematic review	11 (26.8)
Scoping review	10 (24.4)
Other (e.g., critical, rapid, realist, narrative)	20 (48.8)
Data collection/source(s)^a^	
Review articles (*n* = 41; 15.4%)	
Published research articles	30 (73.2)
Published research and gray literature	8 (19.5)
Other (e.g., research articles plus expert panel)	3 (7.3)
Non-review Articles (*n* = 226; 84.6%)	
Survey (web, paper, researcher-administered, multiple formats, unclear)	83 (36.7)
Interviews and/or focus groups	77 (34.1)
Administrative data (e.g., criminal records, health records)	32 (14.2)
Documents/media	6 (2.7)
Social networking data	3 (1.3)
Other/multiple	25 (11.1)
Sample (non-review articles)^b^	
Survivors	73 (32.3)
Mixed survivors (some victims, some not)	18 (8.0)
Perpetrators	31 (13.7)
Mixed perpetrators (some perpetrators, some not)	3 (1.3)
Both (survivors and perpetrators)	12 (5.3)
Mixed both (some victims and perpetrators, some neither)	13 (5.8)
Multiple (more than one sample/study)	11 (4.9)
IPV-sector providers	20 (8.8)
Other providers (e.g., police, lawyers, physicians)	12 (5.3)
Mixed providers	14 (6.2)
Other (e.g., general samples, families, policy documents)	19 (8.4)

*Note.* IPV = Intimate partner violence.

a Percent use the *n* for their category as the denominator.

b Percent use the total number of non-review articles, 226, as the denominator.

Many articles did not specify or were unclear regarding the language used for data collection (*n* = 148, 55.4%). Overall, most articles collected data in English only (*n* = 160, 60.0%), or likely collected data in English (determined by factors such as where in Canada the research took place, participant quotes presented in English with no mention of translation, authors being from predominantly English-speaking institutions, etc.). Only 6.7% (*n* = 18) of the articles collected data exclusively in French, and 9.4% collected (or likely collected) data in both official languages (*n* = 25). The language of data collection for other articles was either English plus at least one other language (other than French; *n* = 5, 1.9%), both official languages plus at least one other (*n* = 3, 1.1%), only other non-official languages (*n* = 2, 0.7%), or the language could not be clearly determined (*n* = 54, 20.2%).

### Author Characteristics and Research Locations

The average number of authors on an article was 4.69 (*SD* = 3.37), and relatively few (*n* = 18, 6.7%) had more than eight (range = 1–35). Overall, there were 183 unique first authors for all included articles. Of these, 74.9% (*n* = 137) had one first author paper included, 23.0% (*n* = 42) had two to four articles included, and 2.2% (*n* = 4) had five or more articles included. Many of these first authors were also contributing authors on other included articles. The vast majority of included articles had a Canadian-affiliated first author (*n* = 246, 92.1%). These articles represented 162 unique Canadian-affiliated first authors, 45 (27.8%) of which had more than one included article. Of the articles with a Canada-affiliated first author, 33.7% (*n* = 83) were led by a trainee, defined as an individual labeled as a student or postdoctoral fellow (e.g., in their author bio) and/or a person without a PhD (or MD) at the time of publication. Most articles had only Canada-affiliated authors (*n* = 217,^4^ 81.3%). However, among articles involving international collaborations (*n* = 50, 18.7%), four countries were represented in five or more articles: the United States (*n* = 27, 54.0%), Australia (*n* = 15, 30.0%), the United Kingdom (*n* = 9, 18.0%), and New Zealand (*n* = 5, 10.0%). Overall, Canada-affiliated authors had collaborated with authors from 25 other countries, and an average of 1.78 (*SD* = 1.38) countries.

With respect to where the research was conducted, most articles reported collecting data in one Canadian province or territory only (*n* = 149, 55.8%). The other articles reported collecting data in two to five provinces/territories (*n* = 18, 6.7%), six or more provinces/territories (*n* = 17, 6.4%), did not specify where in Canada data were collected (*n* = 65, 24.3%), or something else (*n* = 18, 6.7%), such as reviews that did not specify the country of included studies or studies involving social networking data likely to include some Canadian data but not specifying participants’ country of residence (e.g., Reddit posts). Of the articles that clearly specified where the research occurred in Canada (*n* = 180), the most common location was Ontario (*n* = 82, 45.6%). Among articles specifying a single location of data collection in Canada (*n* = 149), studies occurred in Ontario (*n* = 54, 36.2%), Quebec (*n* = 38, 25.5%), Alberta (*n* = 13, 8.7%), British Columbia (*n* = 13, 8.7%), Saskatchewan (*n* = 13, 8.7%), Manitoba (*n* = 4, 2.7%), New Brunswick (*n* = 3, 2.0%), or in a province/territory that was not clearly specified (*n* = 11, 7.4%). Studies that collected data from six or more provinces and territories had often accessed a government data set, such as the General Social Survey, which had its data used in nine of our included studies.

### Research Funding

Analysis of funding sources indicated that, overall, a third (33.7%, *n* = 90) of included articles reported no funding source. Of the 177 articles with funding, 32.2% (*n* = 57) reported more than one source of funding. The Social Sciences and Humanities Research Council of Canada (SSHRC) was the most common funder, with over a quarter (42.9%, *n* = 76) receiving funding from this source. Few articles indicating funding received this from sources outside of Canada (6.8%, *n* = 12). Of note, the two federal government departments with specific GBV/IPV funding initiatives in place were cited as funding less than 6% (WAGE = 2.8%, PHAC = 2.8%) of the funded studies. In terms of accessibility, 42.7% (*n* = 114) of all included articles were available via open access publication models.

### Emerging and/or Innovative Topics and Methods

Finally, we assessed the overall set of included articles to flag those that either begin to address known gaps in Canadian (and global) IPV research or present innovative and/or emerging approaches to topics or methodologies that could advance our thinking in the field (Table 3). These included: (a) understanding predictors and experiences of IPV as patterned and using appropriate instruments and statistical techniques to assess these patterns; (b) addressing specific gaps in knowledge of IPV experiences and barriers to care in under-served groups, especially among Indigenous and 2SLGBTQ+ people; and (c) explicitly embedding equity considerations in the design and assessment of IPV interventions.

**Table 3.** Innovative and Emerging Topics in Included Articles.

Area of Innovation	Gap or Problem Addressed	Description of Research	Included Article
Patterns and/or profiles of IPV indicators and experiences	Measurement, especially cross-sectional, does not capture the complexity of people’s experiences, often leading to misrepresentations.	Used cluster analysis to identify risk factor clusters for domestic homicide.	Dawson and Piscitelli (2021)
		Used latent class analysis to identify four classes of men’s IPV experiences based on severity.	Lysova and Dim (2022)
		Used the CASr-SF to measure IPV including new scoring method that accounts for severity and frequency.	Mantler, Shillington et al. (2022)
		Used cluster analysis to identify profiles of barriers to support for victims of domestic homicide.	Musielak et al. (2020)
Research with under-served groups	Few studies include samples from Northern and/or Indigenous settings or 2SLGBTQ+ communities, each of which has unique IPV-related needs and experiences.	Interviewed providers in Northern communities about their experiences and women’s unique IPV needs and barriers.	Faller et al. (2021)
		Examined the psychometric properties of IPV items by sex assigned at birth and sexual orientation.	Yakubovich et al. (2022)
Equity-promoting interventions	Few studies examine interventions that specifically address the needs of equity-seeking populations, including those with past or ongoing experiences of structural violence.	Interviewed staff about providing safe, equitable and trauma-informed virtual IPV interventions during the COVID-19 pandemic.	Ghidei et al. (2022)
		Systematic review of reviews assessing IPV intervention for inclusion of trauma- and violence-informed and equity-promoting approaches.	Wathen and Mantler (2022)

*Note.* CASr-SF = Composite Abuse Scale Revised-Short Form (Ford-Gilboe et al., 2016); IPV = Intimate partner violence.

## Discussion

This scoping review examined the type and extent of recent IPV research in Canada. The jurisdictional scope, following Bradbury-Jones et al. (2019), was used to understand what kind of IPV research is being done in Canada, how this relates to past and ongoing policy priorities represented by government funding initiatives, and where strengths and gaps exist.

In terms of what is being studied, we found a continued focus on descriptions of IPV, especially its prevalence (overall and in some groups), correlates, and consequences. Almost a third of studies described services for survivors (and less so for perpetrators), including service user and provider experiences, but did not evaluate service effectiveness, a finding seen in other systematic reviews (e.g., MacGregor et al., 2021). However, in a historically underfunded service sector (Burnett et al., 2016; Harris et al., 2014), it is crucial to move beyond description and develop evidence of effectiveness, access, and acceptability of interventions for various groups, as these form the foundation for additional resources that will benefit survivors. A recent review of the international IPV intervention literature calls for not only theoretical development and assessment of interventions, but also an explicitly trauma- and violence-informed approach, which attends to both interpersonal and structural forms of violence and how these drive inequities in access to services, and health and social outcomes (Wathen & Mantler, 2022); we saw this in some, but not many, included papers. COVID-19 was a topic in approximately 10% of included studies, which, given the time needed to conduct research and publication lags, indicates that researchers were able to quickly pivot to examining pandemic impacts on IPV survivors and services; we anticipate this topic gaining significant prominence in the literature following our end search date in 2022.

In terms of methodology, we found a good mix of quantitative, qualitative, and, importantly, mixed methods designs, across types of research questions studied, including intervention work. We also noted some evolution in measurement away from the CTS and toward instruments that capture more patterned experiences (e.g., Composite Abuse Scale, Hegarty et al., 2005; and CASr-SF, Ford-Gilboe et al., 2016)—a key concern as we attempt to (re)integrate a gendered, theoretically grounded understanding of IPV in research (M. P. Johnson, 2011). That said, about 40% still used the CTS or an adaptation of it (see H. Johnson, 2015 for a discussion of the CTS’s limitations, especially regarding gender), and only about half of the articles that reported measuring IPV used or adapted a validated measurement tool. About a third of articles were assessed as having a theoretical grounding; of those, fewer than 40% were explicitly feminist, though many of these were critically oriented in some way. We did note some advance in statistical techniques to better highlight the patterned nature of IPV indicators and experiences. Our findings in this respect are similar to those of Bradbury-Jones et al. (2019), who found it difficult to locate European articles about GBV as many did not explicitly use the term “gender” in titles or abstracts. Clearly, there is still work to be done in designing and implementing IPV studies that integrate theory, methodology, and method in ways that align with the root causes of IPV and ethically and accurately assess causes, consequences, and potential interventions to improve survivor outcomes (Sánchez-Prada et al., 2023; Wathen & Mantler, 2022).

With respect to the relative quantity of IPV research, in their one-year snapshot limited to specific journals, Bradbury-Jones et al. (2019) identified only 32 GBV articles across all of Europe, which has a population almost 20 times higher than Canada; our 3-year time period located 267 IPV-specific studies, indicating that Canadian researchers make an important contribution to world-wide IPV literature. This may be related to GBV and IPV being articulated as federal, and in some cases provincial/territorial, priorities.

In terms of who is doing Canadian IPV research, the numbers were robust, with the 267 analyzed articles having 162 unique Canada-affiliated first authors, and an average of just under five authors per paper. Even accounting for overlap, the included articles represent an impressive cadre of IPV researchers in Canada and our finding that about a third of Canadian first authored publications were trainee-led points to a positive future regarding capacity for ongoing IPV research in Canada. Importantly, our review also identified breadth in terms of where in Canada research was located but still a preponderance of English-language publications (95%), which may be due primarily to the lack of relevant French-language journals and that francophone authors often publish in English. We also noted strong international collaborations, with authors from 25 countries outside of Canada identified as collaborators.

However, certain groups are still under-represented in Canadian IPV studies. Gender and sexual minority people, who account for over 1 million Canadians (Statistics Canada, 2022), were included in less than a quarter of non-review studies. We noted that authors, especially of quantitative studies, reported that they attempted to analyze data from gender and sexual minority groups, but too few participated to ensure statistical power. We examine gender and sexual orientation in IPV research in a companion review paper (MacGregor et al., under revision). Similarly, only 4% of papers included data specific to Indigenous groups, which is low given the over-representation, due to historical and structural conditions, of Indigenous women in national violence statistics (Cotter, 2021; Women and Gender Equality Canada, 2022b). We also noted that less than 5% of papers included data from rural women/survivors, who are also under-served (DeKeseredy et al., 2020). Finally, still more work is needed to understand men’s experiences of IPV (Scott-Storey et al., 2022) and experiences of newcomer, racialized, disabled, and minority language communities in Canada.

We found few articles that examined IPV (or GBV) policies and their impacts, and few (<6%) that indicated funding directly from federal government sources with violence-specific funding initiatives (i.e., WAGE and PHAC); while these initiatives were broad in their scope and not specifically directed at researchers, research and evaluation were funded. It should also be noted that in some cases, federal funds were directed to Canadian research funding agencies (e.g., WAGE co-sponsoring research initiatives with SSHRC); thus, some of the articles that indicated funding by SSHRC, Canadian Institutes of Health Research, or other federal research councils (42.3%) may have been indirectly funded by these federal initiatives.

### Limitations and Future Research

To provide a snapshot of Canadian IPV research at a time when several key factors were driving the need for more, and better, evidence (i.e., high prevalence, costly impacts, government response and exacerbation due to the global pandemic), we made several decisions about the scope of the review that may have impacted our findings. First, we excluded gray literature, which may have missed many outputs of non-academic research and evaluation. Our forthcoming companion analysis of knowledge mobilization strategies for Canadian IPV research is intended to identify additional products arising from these federal, and other, initiatives. We also limited our topic to IPV research, instead of GBV more broadly, both to understand what was happening in this specific area, but also to keep the scope manageable. In terms of pressing gaps outside of more theoretically-grounded and equity-promoting intervention work, future research must address the under-representation of groups and areas facing marginalization in studies (but over-representation in victimization data), including Indigenous groups, Black and other people of color, people with disabilities, and those living in rural and remote areas. Research in Canada’s three northern Territories, under-represented in our data, would help address some of these gaps.

## Conclusions

Overall, we identified an impressive amount of Canadian IPV research published in the first 3 years of the 2020s. The work was conducted by a range of interdisciplinary scholars, using various designs and methodologies, with a strong emerging cadre of young researchers, and robust international collaborations. However, there remains an overfocus on IPV’s epidemiology and on descriptions of service use, and (though emerging) a lack of research examining the effectiveness and implementation of interventions, especially those grounded in theoretical, gendered, and trauma- and violence-informed frameworks. Related to this, there remains an emphasis on cis-gender and heterosexual experiences of IPV, especially among younger White women in Canada. There is an ongoing and urgent need for intervention research specifically examining the experiences of gender and sexual minority people, Indigenous Peoples, racialized groups, those with various abilities, and newcomers. Funders and researchers are encouraged to consider moving resources from ongoing description of well-established factors to implementation, scale-up and continuous assessment of evidence-informed interventions, and, crucially, primary prevention of IPV and GBV.

## Critical Findings

- Canada-affiliated researchers have contributed significantly to the field of IPV in recent years, with 267 peer-reviewed articles published from 2020 to 2022.
- Across the 50 included articles involving international collaborations, Canada-affiliated authors had collaborated with authors from 25 different countries.
- The most common topics studied were IPV (and related) services (e.g., barriers to services) and IPV and psychological factors (e.g., mental health correlates of IPV).
- Overall, there remains an overfocus on IPV’s epidemiology and descriptions of service use, and not enough research examining the effectiveness and implementation of robust, theoretically-grounded interventions.

## Implications for Practice, Policy, and Research

- More mixed-method research is needed to assess theoretically-grounded and equity-promoting IPV interventions as well as the experiences of the following understudied groups: Indigenous people, 2SLGBTQ+ people, Black and other people of color, women/survivors in rural/remote areas, men, newcomers, people with disabilities, and minority language communities in Canada.
- Policymakers and funders should consider directing funding and policy attention to interventions that explicitly reduce inequities in IPV experiences and outcomes, while being trauma- and violence-informed to ensure safety and well-being of victim/survivors, and staff providing care.
- Service organizations implementing existing and innovative interventions are encouraged to partner with researchers to evaluate the effects of these activities, while those funding services should recognize the importance of quality improvement and provide funding for evaluation work.

## Notes

1. For example, see the Global Database on Violence Against Women at https://evaw-global-database.unwomen.org/en.

2. Articles published in 2023 were eligible if the advanced online version was released in 2020 to 2022; similarly, advanced online 2019 publications were included if their final publication date was within the eligible date range.

3. Review articles were coded as not measuring IPV and were, therefore, not included in this analysis, although the articles they reviewed may have measured IPV.

4. Two of these articles included one author with a Canadian affiliation plus an affiliation in another country.
